# In Vitro and In Silico Studies on the Anti-H1N1 Activity of Bioactive Compounds from Marine-Derived *Streptomyces ardesiacus*

**DOI:** 10.3390/md23040149

**Published:** 2025-03-29

**Authors:** Yung-Husan Chen, Cheng-Yang Hsieh, Chun-Tang Chiou, Engelo John Gabriel V. Caro, Lemmuel L. Tayo, Po-Wei Tsai

**Affiliations:** 1 Xiamen Key Laboratory of Natural Products Resources of Marine Medicine, Xiamen Medical College, Xiamen 361023, China; cyxuan@xmmc.edu.cn; 2 Fujian Provincial University Marine Biomedical Resources Engineering Research Center, Xiamen Medical College, Xiamen 361023, China; 3 Department of Food Science, National Taiwan Ocean University, Keelung 202, Taiwan; d339108001@tmu.edu.tw; 4 Department of Chemical and Materials Engineering, National I-Lan University, Yilan 260, Taiwan; 5 National Research Institute of Chinese Medicine, Ministry of Health and Welfare, Taipei 112, Taiwan; ctchiou@nricm.edu.tw; 6 School of Chemical, Biological, and Materials Engineering and Sciences, Mapúa University, Manila 1002, Philippines; ejgvcaro@mymail.mapua.edu.ph; 7 Department of Biology, School of Health Sciences, Mapúa University, Makati 1200, Philippines

**Keywords:** 1-acetyl-β-carboline, Influenza A, network pharmacology, molecular docking, marine-derived bioactive compounds

## Abstract

This study explores the potential anti-H1N1 Influenza A activity of bioactive compounds extracted from *Streptomyces ardesiacus*, a marine-derived microorganism known for producing diverse secondary metabolites. Four major compounds—1-acetyl-β-carboline, 1*H*-indole-3-carbaldehyde, anthranilic acid, and indole-3-carboxylic acid—were isolated and characterized through NMR. Among these, the identified structure of 1-acetyl-β-carboline showed the highest IC_50_ effect, with a dose of 9.71 μg/mL in anti-influenza assays. Using network pharmacology and molecular docking analyses, the interactions of these compounds with key proteins involved in H1N1 pathogenesis were examined. Protein–protein interaction (PPI) networks and Gene Ontology enrichment analysis revealed CDC25B, PARP1, and PTGS2 as key targets, associating these compounds with pathways related to catalytic activity, inflammation, and cell cycle regulation. The molecular docking results demonstrated that 1-acetyl-β-carboline exhibited binding affinities comparable to Tamiflu, the positive control drug, with LibDock scores of 81.89, 77.49, and 89.21 for CDC25B, PARP1, and PTGS2, respectively, compared to Tamiflu’s scores of 84.34, 86.13, and 91.29. These findings highlight the potential of the active compound 1-acetyl-β-carboline from *S. ardesiacus* as a novel anti-influenza agent, offering insights into their molecular mechanisms of action. The results support further in vitro and in vivo studies to validate the observed inhibitory mechanisms and therapeutic applications against H1N1 Influenza A.

## 1. Introduction

The genus *Streptomyces*, a prominent member of the Actinobacteria phylum, constitutes the largest group within the order Actinomycetales. Morphologically, its growth, branching, and development at the tips of its hyphae exhibit characteristics similar to those of filamentous fungi, underscoring its significance in microbial ecology and biotechnology [1,2]. *Streptomyces* species are ubiquitous, thriving in both terrestrial and aquatic ecosystems, where they play a critical role in organic matter decomposition and nutrient cycling. More importantly, they exhibit extensive secondary metabolic activity, producing a diverse range of biologically active metabolites, which are pivotal to pharmaceutical research. These secondary metabolites include a vast repertoire of antibiotics, accounting for nearly two-thirds of the naturally derived antibiotics currently utilized in clinical practice [3,4]. Notable examples include actinomycin, streptomycin, tetracycline, neomycin, viridomycin, kasugamycin, and kanamycin, which have been widely employed to combat bacterial infections [3,4,5].

Due to their remarkable ability to synthesize a broad spectrum of bioactive compounds and enzymes, *Streptomyces* species have been extensively commercialized and applied in diverse biotechnological and pharmaceutical industries [6]. As a prolific source of novel therapeutic agents, *Streptomyces* continues to garner significant scientific interest. However, despite decades of research, many of their biochemical pathways and metabolic potentials remain largely unexplored [7]. Recent genomic sequencing and bioinformatics analyses have revealed that the secondary metabolite biosynthetic potential of marine-derived *Streptomyces* has been significantly underestimated, highlighting an untapped resource for drug discovery [8]. Given the unparalleled ecological diversity of marine microorganisms, genomic and bioinformatic approaches have become essential tools for investigating their metabolic capacities [9,10]. Consequently, marine-derived microbial resources are increasingly recognized as crucial assets in biotechnology, environmental remediation, and public health research [6,10,11,12,13,14,15,16].

*Streptomyces ardesiacus* is a species of actinobacteria known for its diverse secondary metabolites, among which the indole skeleton is a crucial heterocyclic structure found in many bioactive natural compounds [17,18,19]. This bacterium can biosynthesize various indole-based compounds, such as indole alkaloids and tryptophan-derived derivatives, which exhibit significant biomedical value [18,20]. Notably, these indole skeleton derivatives demonstrate anti-inflammatory, antiviral, and antibacterial effects, as well as neuro-therapeutic properties, making them promising candidates for novel drug development [17,18,21,22,23]. Furthermore, these secondary metabolites play critical roles in regulating cellular signaling pathways, microbial community dynamics, and environmental adaptation [22,24,25]. Therefore, *S. ardesiacus* presents substantial pharmaceutical and biotechnological potential in the research and application of indole-based natural products. Beta-carbolines (β-carbolines) are a class of bioactive alkaloids characterized by a pyridoindole skeleton, exhibiting a wide range of pharmacological activities [25]. *S. ardesiacus*, through its secondary metabolic pathways, is capable of producing β-carboline compounds, which hold great significance in the medical field [26,27,28,29,30]. Specifically, these β-carboline derivatives display antitumor, antitubercular, antimalarial, sedative, hypnotic, anticonvulsant, antimicrobial, and antiviral properties [27,31]. Research has shown that certain β-carbolines can inhibit monoamine oxidase (MAO), thereby influencing neurotransmission and offering potential applications in the treatment of neurodegenerative diseases and depression [32,33,34,35]. Additionally, their antimicrobial and antiparasitic activities make them strong candidates for the development of antibiotics and anti-infective drugs [36,37]. As a result, *S. ardesiacus* provides valuable pharmacological resources in the biosynthesis and application of β-carboline compounds, with the potential to drive innovation in drug discovery.

As part of our ongoing research on marine-derived bioactive compounds, we have successfully isolated the major compounds from *S. ardesiacus*. Following culture supernatant extraction and fractionation, four bioactive compounds were identified and subsequently characterized using spectroscopic methods. LCMS was also employed to identify the metabolites. In this study, in silico approaches, including network pharmacology and molecular docking, were employed to evaluate the potential biological activities and mechanisms of action. Given the critical role of preliminary computational analysis in modern drug discovery, network pharmacology and molecular docking serve as essential tools in guiding further experimental validation before progressing to in vivo studies.

## 2. Results

### 2.1. Structural Elucidation of Compounds Isolated from S. ardesiacus

**Figure 1:**
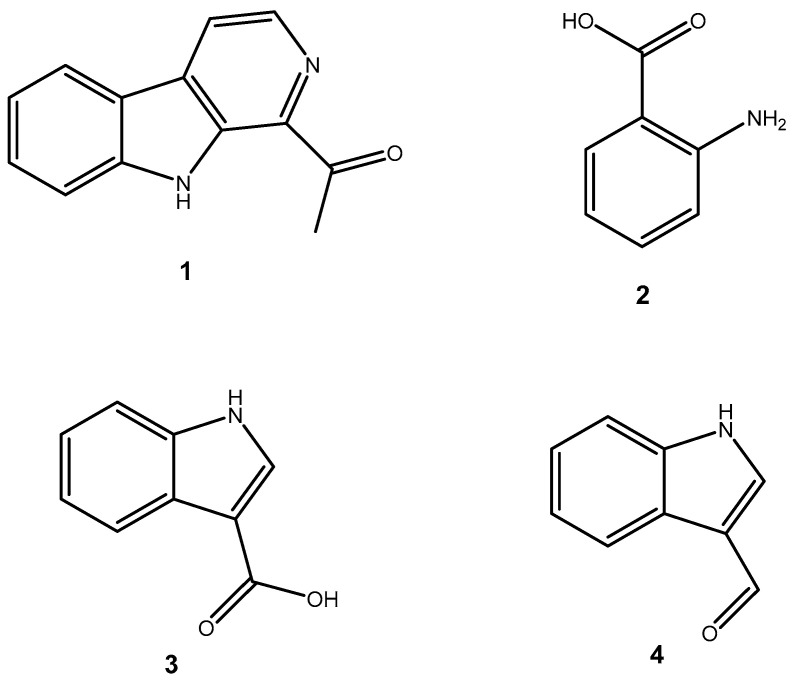
Structures of secondary metabolites **1**–**4** isolated from *S. ardesiacus*.

Figure 1 shows that the chemical structures of compounds **1** to **4** were isolated from *S. ardesiacus*. Compound **1** (1-acetyl-β-carboline): It was identified as the known compound 1-acetyl-β-carboline based on comparisons of its spectra with the previous report, including NMR (^1^H and ^13^C) and mass spectra (Figures S1–S3) [38]. Fraction Fr.3.6.1.1 (1.8 g) was subjected to gradient elution on a silica gel column. Thin-layer chromatography (TLC) analysis of the eluted fractions identified seven subfractions, one of which (Fr.3.6.1.1.1, 41.6 mg) yielded **1** after preparative TLC under the conditions of dichloromethane/petroleum ether = 2:1, with an *Rf* value of 0.59. Given that the initial extract weighed 5.3127 g, the yield of **1** was calculated to be 0.78%.

Compound **2** (anthranilic acid): Fraction Fr.3.6.1.1.3 (267.9 mg) was subjected to preparative TLC using a dichloromethane-methanol system (15:1), followed by purification via high-performance liquid chromatography (HPLC), yielding 2 (128.4 mg). Compound **2** was identified as the known anthranilic acid through comparisons of its NMR (^1^H and ^13^C) and mass spectra (Figures S4–S6) [39].

Compound **3** (indole-3-carboxylic acid): Fraction Fr.3.6.2 was analyzed by TLC (dichloromethane/methanol = 10:1) and purified using preparative TLC, yielding Compound 3 (5 mg). The purity of Compound **3** was assessed using HPLC. Compound **3** was identified as indole-3-carboxylic acid based on comparisons of its spectra with previous reports (Figures S7–S9) [40].

Compound **4** (1*H*-indole-3- carbaldehyde): Fraction Fr.3.6.3 was purified using semi-preparative HPLC on a Welch XB-C18 column (10 × 250 mm, 5 μm) with an isocratic mobile phase of methanol (0.1%), formic acid (45%), and water (55%). The flow rate was 1.5 mL/min, and the retention time was 25.1 min, yielding Compound **4** (3.1 mg). Compound **4** was identified as the known compound based on comparisons of its ^1^H NMR and mass spectra (Figures S10 and S11) [41].

### 2.2. Anti-Influenza Activity

The extracts isolated from *S. ardesiacus* contain four compounds: 1-acetyl-β-carboline, 1*H*-indole-3-carbaldehyde, anthranilic acid, and indole-3-carboxylic acid. Table 1 presents the anti-influenza activity of these compounds. Their IC_50_ (μg/mL) values were 9.71, ND, 82.06, and 81.49, respectively, indicating that 1-acetyl-β-carboline has the highest anti-influenza activity, with an IC_50_ of 9.71 μg/mL.

### 2.3. Network Pharmacology

**Figure 2:**
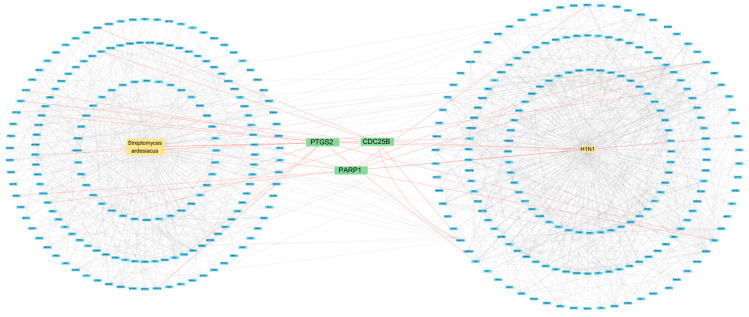
Protein–protein interaction between *S. ardesiacus* isolates and H1N1 Influenza A.

The *S. ardesiacus* extracts contain four compounds: 1-acetyl-β-carboline, 1*H*-indole-3-carbaldehyde, anthranilic acid, and indole-3-carboxylic acid. These were investigated for their correlation with H1N1 Influenza A. The results show that 186 genes were correlated with *S. ardesiacus* extracts, and 194 genes were correlated with H1N1 Influenza A, with a confidence threshold of 0.9 for the highest confidence interactions. Topological analysis, which evaluates the network structure and identifies key nodes based on metrics such as degree, closeness, and betweenness, was performed. The degree, closeness, and betweenness values of 3.82, 7.54, and 23.17, respectively, indicated that three major proteins—CDC25B, PARP1, and PTGS2—are involved in the interaction between microbial isolates and H1N1 Influenza A (Figure 2).

**Figure 3:**
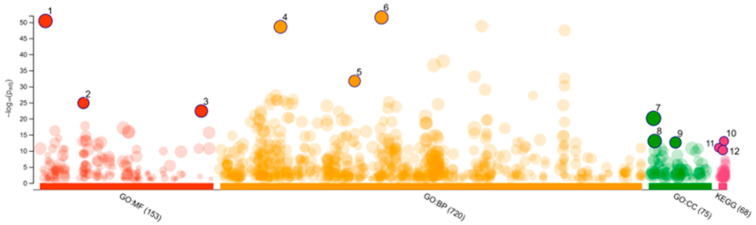
Gene Ontology and KEGG analysis.

Gene Ontology was then applied for term enrichment analysis, and the top three terms for Molecular Function (MF) were catalytic activity, oxidoreductase activity, and catalytic activity acting on a protein; the top three terms for Biological Processes (BPs) were response to chemical, response to organic substance, and response to lipid; the top three terms for Cellular Compartments (CCs) were the cytoplasm, cytosol, and somatodendritic compartments (Figure 3 and Table 2). The KEGG pathway analysis reveals that the top three pathways were serotonergic synapse, steroid hormone biosynthesis, and apoptosis (Figure 3 and Table 2). Based on the STRING analysis of correlation genes between SwissTargetPrediction and the H1N1 data from GeneCards, the results highlight the major pathways involved in the molecular mechanisms of *S. ardesiacus* isolates in relation to H1N1 Influenza A. These results highlight the major pathways involved in the molecular mechanisms of *S. ardesiacus* isolates in H1N1 Influenza A.

### 2.4. Molecular Docking

The topological analysis results of the three major proteins, CDC25B, PARP1, and PTGS2 (Figure 2), were investigated for their correlation with H1N1 Influenza A. The LibDock scores (Table 3) indicate that **1** interacts with proteins CDC25B, PARP1, and PTGS2, with scores of 81.89, 77.49, and 89.21, respectively, while the positive control drug, Tamiflu, has scores of 84.34, 86.13, and 91.29. This suggests that the *S. ardesiacus* isolates exhibit a similar inhibitory effect to the positive drug.

**Figure 4:**
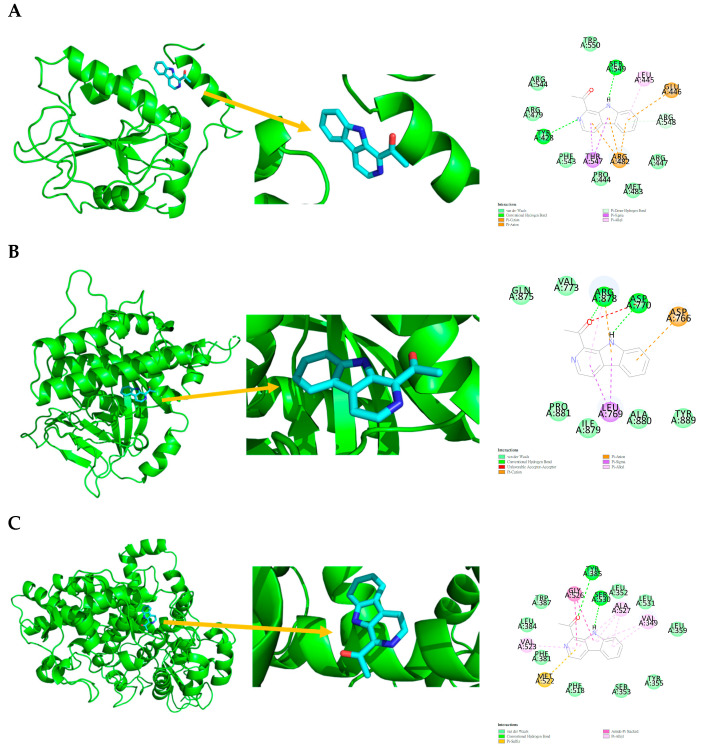
Ligand binding sphere and two-dimensional display of the binding interactions of CDC25B (4WH7), PARP1 (4RV6), and PTGS2 (5KIR) with 1. (**A**) CDC25B (4WH7) and 1; (**B**) PARP1 (4RV6) and 1; (**C**) PTGS2 (5KIR) and 1. A represents the interaction chains; the numbers represent the amino acid sequence numbers.

**Figure 5:**
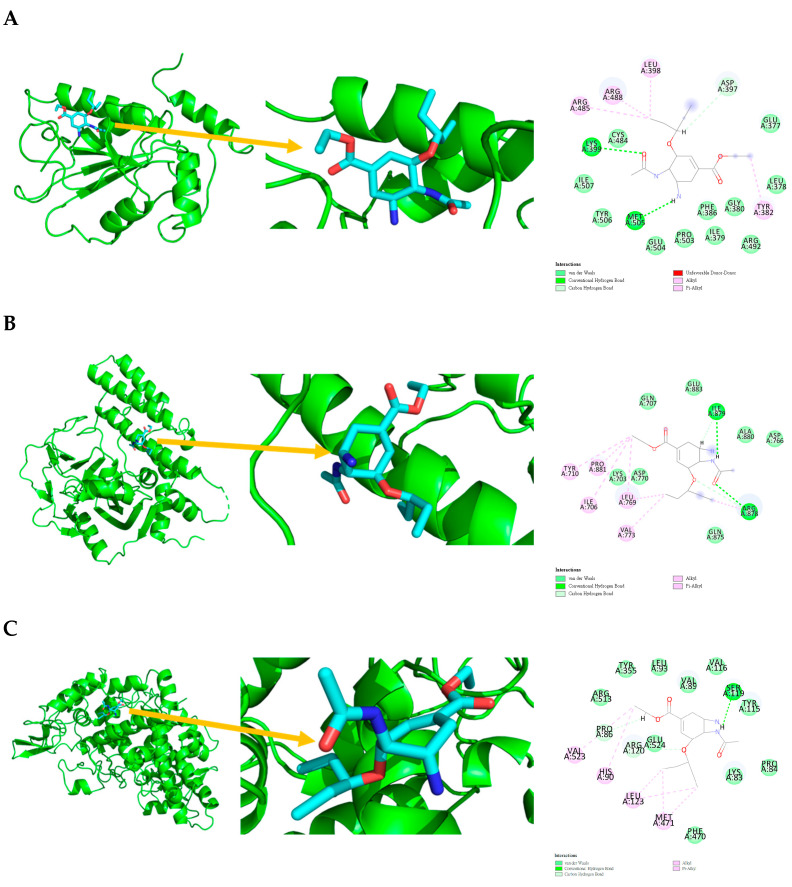
Ligand binding sphere and two-dimensional display of the binding interactions of CDC25B (4WH7), PARP1 (4RV6), and PTGS2 (5KIR) with Tamiflu. (**A**) CDC25B (4WH7) and Tamiflu; (**B**) PARP1 (4RV6) and Tamiflu; (**C**) PTGS2 (5KIR) and Tamiflu. A represents the interaction chains; the numbers represent the amino acid sequence numbers.

To examine the interactions based on the LibDock scores, only **1** showed similar results to Tamiflu with CDC25B, PARP1, and PTGS2. A two-dimensional display of the ligand binding sphere and protein interactions was also analyzed. This study investigates the interactions of **1** and Tamiflu with key proteins such as CDC25B, PARP1, and PTGS2, with detailed binding interactions summarized in Table 4. The 2D and 3D interactions between **1** and the targets CDC25B, PARP1, and PTGS2 are shown in Figure 4 and Table 4. Similarly, the 2D and 3D interactions between the positive control drug Tamiflu and the targets CDC25B, PARP1, and PTGS2 are shown in Figure 5 and Table 5.

Compound **1** interaction with CDC25B (4WH7) is shown in Figure 4 and Table 4, with van der Waals interactions with PRO444, ARG447, ARG479, MET483, PHE543, ARG544, ARG548, and TRP550; conventional hydrogen bonds with TYR428 and SER549; pi-donor hydrogen bonds with ARG548; pi-sigma interaction with THR547; pi-alkyl interaction with LEU445; and pi-cation and pi-anion interactions with GLU446 and ARG482.

Compound **1** interaction with PARP1 (4RV6) is shown in Figure 4 and Table 4, with van der Waals interactions with VAL773, GLN875, ILE879, ALA880, PRO881, and TYR889; conventional hydrogen bonds with ASP770 and ARG878; pi-sigma interaction with LEU769; pi-alkyl interaction with ARG878; pi-cation and pi-anion interactions with ASP766 and ARG878; and an unfavorable acceptor–acceptor interaction with ASP770.

Compound **1** interaction with PTGS2 (5KIR) is shown in Figure 4 and Table 4, with van der Waals interactions with LEU352, TYR355, LEU359, LEU384, TRP387, SER353, LEU531, and PHE518; conventional hydrogen bonds with TYR385 and SER530; pi-sulfur interaction with MET522; pi-alkyl interactions with VAL349, VAL523, and ALA527; and amide-pi stacking with GLY526.

Tamiflu interaction with CDC25B (4WH7) is shown in Figure 5 and Table 5, with van der Waals interactions with GLU377, LEU378, ILE379, GLY380, PHE386, CYS484, ARG492, PRO503, GLU504, TYR506, and ILE507; conventional hydrogen bonds with LYS399 and MET505; carbon-hydrogen bonds with ASP397; and pi-alkyl and alkyl interactions with TYR382, LEU398, ARG485, and ARG488.

Tamiflu interaction with PARP1 (4RV6) is shown in Figure 5 and Table 5, with van der Waals interactions with LYS703, GLN707, ASP766, ASP770, GLN875, ALA880, and GLU883; carbon-hydrogen bonds with ARG878; conventional hydrogen bonds with ILE879 and ARG878; and pi-alkyl and alkyl interactions with ILE706, TYR710, LEU769, VAL773, and PRO881.

Tamiflu interaction with PTGS2 (5KIR) is shown in Figure 5 and Table 5, with van der Waals interactions with LYS83, PRO84, VAL89, LEU93, TYR115, VAL116, TYR355, PHE470, ARG513, and GLU524; conventional hydrogen bonds with SER119; carbon-hydrogen bonds with PRO86 and ARG120; and pi-alkyl and alkyl interactions with HIS90, LEU123, MET471, and VAL523.

### 2.5. Molecular Dynamics

Molecular dynamics using GROMACS [42] was conducted to further assess the structural stability of the ligand–receptor complex for both Compound **1** and Tamiflu across all three receptors, PARP1, CDC25B, and PTGS2. The former was the test compound, and the latter was set as the reference ligand as a baseline comparison. Root Mean Square Deviation (RMSD), radius of gyration, backbone root mean square fluctuation (RMSF), and total number of hydrogen bonds between protein and ligand were determined as shown in Figures S12–S15, respectively.

The Kolmogorov–Smirnov test [43] was used to determine the normality of the distributions, through which it was determined that the frequency distributions for all results are not normal distributions. Following this discovery, Cliff’s Delta [44] and Mann–Whitney U testing [45] were conducted to evaluate the degree of effect size and median shift, respectively.

The RMSD of both CDC25B (Cliff’s Delta = −0.6806, MW *p* < 0.0001) and PTGS2 (Cliff’s Delta = −0.5476, MW *p* < 0.0001) exhibited significantly lower values compared to the reference, indicating increased structural stability, whereas PARP1 (Cliff’s Delta = −0.0856, MW *p* < 0.0001) showed only a slight decrease. The radius of gyration results demonstrated that only CDC25B (Cliff’s Delta = −0.3638, MW *p* < 0.0001) showed a significant difference between the drug and reference; on the other hand, PARP1 (Cliff’s Delta = 0.1411, MW *p* < 0.0001) and PTGS2 (Cliff’s Delta = 0.0043, MW *p* = 0.5986) showed small and no meaningful changes, respectively. The RMSF analysis indicates significant differences for CDC25B (Cliff’s Delta = −0.1398, MW *p* = 0.0226) and PTGS2 (Cliff’s Delta = −0.0872, MW *p* = 0.0122), while PARP1 (Cliff’s Delta = −0.0553, MW *p* = 0.2073), again, showed no significant difference between the drug and reference bound complexes. The total number of hydrogen bonds analysis indicated substantial increases in the total number of hydrogen bonds between the drug and receptors for CDC25B (Cliff’s Delta = 0.1643, MW *p* < 0.0001) and PTGS2 (Cliff’s Delta = 0.1411, MW *p* < 0.0001). PARP1 (Cliff’s Delta = −0.0034, MW *p* = 0.3060) once again showed similar results between the drug and the reference.

Table S1 lists all relevant statistical values. In summary, the results from the molecular dynamics indicate that the drug–receptor complexes display equal or greater affinity when compared to the reference ligand, as shown by the results of the Cliff Delta values being mostly negative, very slightly positive, or zero, which implies lower values, thereby indicating equally or more stable structures according to RMSD, radius of gyrations, RMSF, and the total number of hydrogen bonds.

### 2.6. ADMET Analysis

ADMET analysis and druglikeness were evaluated using the SwissADME webserver [46]. The results in relation to absorption, metabolism, and druglikeness are summarized in Tables S2, S3, and S4, respectively.

All potential drugs display high absorption through GI intake methods such as oral administration. It should be noted, however, that all potential drugs are BBB permeant, which could present some problems within the central nervous system. Although the drugs are BBB permeant, none are Pgp substrates, which means potentially low retention within the brain [47], as opposed to Tamiflu, which displays the opposite characteristics for GI absorption and BBB permeability as well. All molecules tested in the study were found to have poor skin permeability, however, as shown by their negative log Kp values [48].

Although all the drugs were found to not inhibit four major cytochrome P450 enzymes, three of the potential drugs are CYP1A2 inhibitors. This could potentially affect medications relating to antidepressants or caffeine metabolism.

Among the compounds under study, only 1-acetyl-β-carboline showed complete obedience to the druglikeness criteria. 1*H*-indole-3-carbaldehyde violated Ghose (MW < 160, number of atoms < 20), Muegge (MW < 200), and leadlikeness (MW < 250) criteria in addition to 1 Brenk alert (aldehyde). Anthranilic acid also violated Ghose (MW < 160, MR < 40, number of atoms < 20), Muegge (MW < 200), and leadlikeness (MW < 250) with 1 Brenk alert (aniline). Indole-3-carboxylic acid violated Ghose (number of atoms < 20), Muegge (MW < 200), and leadlikeness (MW < 250) criteria; no additional structural alerts were raised. The reference compound Tamiflu also had violations in Veber (TPSA > 140), Egan (TPSA > 131.6), Muegge (XLOGP3 < −2, TPSA > 150), and leadlikeness (MW > 350, Rotors > 7), with an additional structural alert by Brenk (phosphor). This indicates that among the potential drugs, 1-acetyl-β-carboline has an extremely high potential for therapeutic use.

## 3. Discussion

Marine environments provide a window into the capabilities of nature. Unique environmental circumstances pressured organisms to develop novel methods for dealing with problems unknown to those on the surface. 1-acetyl-β-carboline isolated from marine *Streptomyces* sp. Compound **1** was found to be a potent anti-methicillin-resistant *Staphylococcus aureus*, possibly illustrating a defense mechanism against antagonistic actions between the two bacterial strains [49]. Indole alkaloids such as 1*H*-indole-3-carbaldehyde (Compound **2**) similarly show antagonistic functions against other bacterial and fungal species [50]. Anthranilic acid (Compound **3**) was determined to be a spore germination inhibitor [51]. Lastly, Indole-3-carboxylic acid (Compound **4**) was found to be a secondary metabolite meant for antimicrobial activity as a form of facilitating symbiosis between other organisms [52]. Regardless of their intended purposes, compounds and metabolites have repeatedly shown high potential as novel treatment strategies for viral, fungal, and oncogenic conditions.

Influenza virus is a global health issue that poses a significant threat to public health worldwide [53,54]. It is often accompanied by complications and can evolve within the host, leading to zoonotic infections, among other issues [53,55,56]. Network pharmacology indicates that CDC25B, PARP1, and PTGS2 are key target proteins involved in the interaction between *S. ardesiacus* isolates and H1N1 Influenza A (Figure 2). The CDC25B protein influences the mitotic cell cycle. In humans, it not only serves as a regulator of CDC2 kinase during mitosis but also activates CDC25 tyrosine phosphatases [57]. Additionally, it can impair viral polymerase activity, thereby inhibiting the replication of influenza virus strains [54,57,58].

Some of the research has confirmed the network pharmacology results. As Figure 2 indicates, there is a correlation between *S. ardesiacus* isolates and H1N1 Influenza A. Influenza viruses often overcome multiple host defense mechanisms and species-specific barriers (such as those in humans, pigs, and avian species) to infect new hosts [59,60]. These barriers include interactions with RNA-dependent RNA polymerase (RdRP) for replication [49]. The host factor PARP1 (poly(ADP-ribose) polymerase 1) modulates chromatin remodeling and the transcription of human, swine, and avian influenza RdRP within human cells [61,62]. Another protein, type I interferon receptor 1 (IFNAR1), enhances the self-propagation of the influenza virus within the host [63,64]. Studies have shown that PARP1 can inhibit viral replication and suppress the degradation of interferon alpha and beta receptor subunit 1 (IFNAR1), thereby exerting an antiviral effect [65,66].

Cyclooxygenase-2 (COX-2), also known as prostaglandin-endoperoxide synthase 2, exists in two isoforms: COX-1 and COX-2. Among them, COX-2 is primarily expressed in inflammatory cells. It converts arachidonic acid, released from membrane phospholipids by phospholipase A2 (PLA2), into prostaglandin H₂ (PGH₂), thereby triggering the body’s inflammatory response [67,68,69,70]. This process plays a critical role in the pathophysiology of conditions such as pain, fever, tumors, and viral infections (e.g., influenza), which can ultimately lead to organ damage and functional impairment [68,69,70,71]. Studies have demonstrated the antimicrobial activity of 1-acetyl-β-carboline, including its synergistic antibacterial effects against MRSA [49,72]. Additionally, 1-acetyl-β-carboline has been shown to significantly inhibit COX-2 expression, with an IC₅₀ value of 1.423 µM [49,72].

In the analysis of 366 disease-related proteins (Figure 1), Gene Ontology and KEGG pathway analyses (Figure 2 and Table 2) reveal that CDC25B is primarily involved in the regulation of the cell cycle, with some relevance to apoptosis. PARP1 serves as a key regulator of DNA repair and plays an important role in responding to chemical stress, apoptosis, and maintaining genomic stability. PTGS2 (also known as COX-2) is closely associated with inflammatory responses, lipid metabolism (particularly the metabolism of arachidonic acid), and the response to chemical signals, playing a significant role in prostaglandin biosynthesis and apoptosis.

The interactions of 1-acetyl-β-carboline and Tamiflu with key proteins such as CDC25B, PARP1, and PTGS2 were thoroughly examined through LibDock scores, as shown in Table 3. The analysis of the two-dimensional ligand binding spheres and protein interactions provided valuable insights into the molecular interactions governing these bindings. The interaction profile of 1-acetyl-β-carboline with CDC25B (4WH7) reveals a complex network of binding forces, including van der Waals interactions, conventional hydrogen bonds, and various aromatic interactions, such as Pi-sigma, Pi-alkyl, and Pi-cation interactions (Figure 3, Table 4) [73,74]. This combination of interactions suggests that 1-acetyl-β-carboline may have a strong affinity for CDC25B, potentially affecting its role in regulating the cell cycle. The similar binding characteristics observed with PARP1 (4RV6)—involving van der Waals forces, hydrogen bonds, and aromatic interactions (Figure 4, Table 4) [75,76]—further indicate that 1-acetyl-β-carboline may interact with the protein in a way that modulates its enzymatic activity, which could have therapeutic implications. On the other hand, the interaction with PTGS2 (5KIR) is distinguished by pi-sulfur and amide-pi stacking interactions (Figure 4, Table 4) [77,78,79,80], which could be significant in understanding how 1-acetyl-β-carboline might affect inflammatory pathways regulated by PTGS2.

Similarly, Tamiflu’s interaction with these proteins presents notable differences and similarities when compared to 1-acetyl-β-carboline. The binding of Tamiflu to CDC25B (4WH7) and PARP1 (4RV6) is predominantly driven by van der Waals interactions and conventional hydrogen bonds, with additional carbon-hydrogen and Pi-alkyl interactions (Figure 5, Table 5) [73,74,81,82]. These results suggest that Tamiflu, much like 1-acetyl-β-carboline, binds tightly to these proteins, potentially influencing their functions in the context of viral replication and immune modulation. Notably, the interaction of Tamiflu with PTGS2 (5KIR) is characterized by van der Waals interactions, hydrogen bonds, and Pi-alkyl interactions (Figure 5, Table 5) [73,74,75,76,81,82], which suggests a similar binding mode to that of 1-acetyl-β-carboline, but with variations in the specific types of interactions. The comparative analysis of 1-acetyl-β-carboline and Tamiflu with CDC25B, PARP1, and PTGS2 indicates that while both compounds interact with these proteins through similar forces, the specific types of interactions differ. These findings highlight the complexity and diversity of ligand binding to these targets, suggesting potential avenues for therapeutic development.

The pursuit of more drugs is a race against time. Viruses, with their high rate of proliferation and evolution, are a problem as the current procedures of drug discovery involve long durations and lots of resources [83]. This highlights the importance of studying metabolites from organisms hailing from unique environments, such as marine environments, due to the unique metabolites found within them [49,50,51,52]. Not only can these metabolites be isolated as-is for medicinal applications, but they may also serve as the basis for modifications and drug re-purposing projects, which can cut down the time required to present potential drugs for particularly difficult-to-treat pathogens.

In this study, we investigated the multitarget effects of various compounds, as each target influences distinct pathways, which may interact with one another within the context of the disease pathway. By combining multiple in silico tools, we were able to predict the target profiles and pharmacological actions of the compounds. However, the study provides only limited evidence supporting these predictions. Consequently, additional in vitro and in vivo experiments are necessary to validate our findings. The in silico methods developed in this study offer novel insights into the mechanisms of action, potentially advancing research in this field.

## 4. Materials and Methods

### 4.1. Instruments and Materials

#### 4.1.1. Experimental Materials

##### Experimental Strains

The *S. ardesiacus* (accession number: 2-1-3-C-ISP2) strain was provided by the Key Laboratory of Marine Medicinal Natural Product Resources at Xiamen Medical College. This strain was originally isolated from mangrove soil in the Zhangjiang Estuary, Zhangzhou, China. The soil sample was collected from the rhizosphere of mangrove plants at a depth of 5–10 cm, with a salinity of approximately 1% and a pH of 7.2. The isolation process involved serial dilution plating on ISP2 agar medium, followed by incubation at 28 °C for 7 days. Colonies with distinct morphological characteristics were selected and further purified by repeated streaking. The strain was identified based on morphological, physiological, and molecular characteristics.

#### 4.1.2. Main Reagents and Instruments

##### Main Reagents

GR-grade methanol, ethyl acetate, dichloromethane, petroleum ether, acetone, and formic acid were purchased from Xilong Chemical Co., Ltd., Shantou, Guangdong, China; chromatographically pure methanol was sourced from Germany’s CNW; methanol-*d*_4_ and DMSO-*d*_6_ were from Macklin.

##### Main Instruments

Analytical balance (BSA224S, Sartorius, Beijing); high-pressure sterilizer (Autoclave-G154D, Zealway, Xiamen, China); AV-600 superconducting nuclear magnetic resonance spectrometer (Bruker, Rheinstetten, Germany); handheld UV lamp (WFH-204B, Shanghai ChiTang Industrial Co., Ltd., Shanghai, China); high-performance liquid chromatograph (Infinity 1260, Agilent, Santa Clara, CA, USA); automatic fraction collector (BS-100A, Shanghai HuXi Analytical Instrument Co., Ltd., Shanghai, China); ultrasonic cleaner (DS-080S, Dongsen Intelligent Technology Co., Ltd., Shenzhen, China.); rotary evaporator (E-10010N, Zhengzhou Great Wall Scientific Industrial and Trade Co., Ltd., Zhengzhou, China); vibrating incubator (ZQZY-C18, Shanghai Zhichu Instrument Co., Ltd., Shanghai, China); low-temperature coolant circulation pump (DLSB-10/20, Zhengzhou Great Wall Scientific Industrial and Trade Co., Ltd.); laminar airflow cabinet (SW-CJ-1D, Shanghai Boxun Industrial Co., Ltd., Shanghai, China).

### 4.2. Isolation Methods

#### 4.2.1. Fermentation Conditions of *S. ardesiacus*

The seed culture medium consisted of ISP2 solid medium (per liter): malt extract powder (10 g), glucose (10 g), yeast extract (4 g), agar powder (18 g), tryptophan (2.5 g), seawater (salinity 30‰), and a pH of 6.8–7.2. The ISP2 liquid medium had the same composition, excluding the agar.

#### 4.2.2. Extraction and Isolation of Metabolites from *S. ardesiacus*

##### Activation of *S. ardesiacus*

Frozen *S. ardesiacus* strains were exposed to UV light for 20 min at room temperature, followed by streaking on ISP2 solid medium and incubation at 28 °C for 3–5 days.

##### Fermentation Cultivation of *S. ardesiacus*

The ISP2 liquid medium, strain culture plates, and consumables were sterilized in a UV cabinet for 20 min. A single colony agar block was transferred into the medium under aseptic conditions and incubated on a shaker at 28 °C and 120 rpm for one week.

##### Extraction of Fermentation Products

The fermentation broth was filtered through gauze to remove the mycelium. The resulting filtrate was adsorbed onto pre-swollen macroporous resin (D101 resin, Qingdao Chemical Co., China) packed in a chromatography column, washed with distilled water, and eluted with methanol. The methanol eluate was concentrated to dryness using rotary evaporation. The dried residue was then dissolved in 100 mL of distilled water, and secondary metabolites were extracted multiple times with ethyl acetate. The combined ethyl acetate layers were concentrated, yielding a crude extract (5.3127 g).

The crude extract was subjected to TLC using a dichloromethane-methanol solvent system. Gradient fractionation on silica gel open-column (60 g, 60–100 mesh; 3 cm diameter × 50 cm length) yielded six fractions (Fr.1–Fr.6). Fraction Fr.3 was further purified using Sephadex LH-20 gel chromatography. The Sephadex gel was pre-soaked in methanol and tightly packed into a chromatography column (2 cm diameter × 70 cm length), equilibrated with methanol. Fr.3 was dissolved in methanol and loaded onto the column. Elution was performed at a flow rate of one drop per six seconds, with eluates collected into 200 tubes (4 mL each). TLC analysis was conducted every three tubes to identify fractions with similar profiles under UV light and staining with sulfuric acid and bismuth iodide-potassium reagent. Similar fractions were pooled and concentrated, resulting in ten subfractions.

Subfraction Fr.3.6 underwent further purification using open-column silica gel chromatography. The silica gel (25 g, 200–300 mesh; 2 cm diameter × 30 cm length) was suspended in dichloromethane and packed into a column, followed by gradient elution with dichloromethane and dichloromethane-methanol systems (25:1, 10:1), and finally with pure methanol. This process yielded four subfractions (Fr.3.6.1–Fr.3.6.4), which were analyzed by HPLC. HPLC conditions included a methanol-0.1% formic acid mobile phase, gradient elution (5–100% methanol), a Welch XB-C18 column (4.6 × 250 mm, 5 μm), a flow rate of 0.8 mL/min, an injection volume of 20 μL, and a column temperature of 30 °C.

### 4.3. Culture Conditions for Influenza Virus and MDCK Cells

The Influenza A/Brisbane/02/2018 (H1N1) was provided by the Taiwan Centers for Disease Control, Ministry of Health and Welfare of Taiwan. Madin-Darby Canine Kidney (MDCK) cells were obtained from the Bioresource Collection and Research Center (BCRC) of the Food Industry Research and Development Institute (FIRDI), Taiwan. MDCK cells were cultured in Dulbecco’s Modified Eagle Medium (DMEM) containing 10% fetal bovine serum (FBS), 100 units/mL of penicillin, and 100 units/mL of streptomycin. For influenza virus infection, cells were treated with the samples in DMEM supplemented with bovine serum albumin and L-1-tosylamido-2-phenylethyl chloromethyl ketone (TPCK), treated with trypsin (Sigma, St. Louis, MO, USA).

### 4.4. Evaluation of Influenza Virus Infection

MDCK cells were initially seeded into a 96-well microplate and incubated in growth medium overnight. The following day, the cells were washed twice with phosphate-buffered saline (PBS) and inoculated with influenza virus at a 0.01 multiplicity of infection (MOI) for one hour. Afterward, the inoculum was removed, and the cells were treated with the tested sample in DMEM containing 0.2% bovine serum albumin and 2 μg/mL L-1-tosylamido-2-phenylethyl chloromethyl ketone (TPCK), followed by treatment with trypsin for 2 days. For the viability test, the cells were fixed with 4% paraformaldehyde and stained with gentian violet.

### 4.5. Network Pharmacology

#### 4.5.1. Protein–Protein Interaction (PPI)

Four major compounds from *S. ardesiacus* were selected for SwissTargetPrediction [84] based on NMR data. The disease H1N1 Influenza A was analyzed using GeneCards [85], and STRING was used to modulate the protein–protein interaction (PPI) network with a minimum required interaction score of 0.9 for the highest confidence interactions. Cytoscape (version 3.10.2) was employed for topological analysis.

#### 4.5.2. Gene Ontology Term Enrichment Analysis

The g:Profiler was used to perform Gene Ontology Term Enrichment analysis of the correlations between H1N1 Influenza A and the four major compounds isolated from *S. ardesiacus*.

### 4.6. Docking Stimulation

#### 4.6.1. Ligand Preparation

The 3D structures of the four compounds identified from the NMR results of *S. ardesiacus* isolates—1-acetyl-β-carboline, 1*H*-indole-3-carbaldehyde, Anthranilic acid, and Indole-3-carboxylic acid—along with the positive control drug Tamiflu, were obtained from PubChem (Table 6) [86].

#### 4.6.2. Molecular Preparation and Docking analysis

The analysis of PPI target gene results led to the selection of four major proteins from the Protein Data Bank: CDC25B (4WH7), PARP1 (4RV6), and PTGS2 (5KIR), to evaluate their potential in combating H1N1 Influenza A. The docking affinity was examined using BIOVIA Discovery Studio 2019 [87,88]. The coordinates and radii of each protein are shown in Table 7.

### 4.7. Molecular Dynamics

#### 4.7.1. System Preparation

Preparation for the production of molecular dynamics was conducted using BioBB Python module v5.0.0 2024.2 [89]. Output .pdb files from the docking simulations of 1-acetyl-beta-carboline and Tamiflu for all 3 receptors: PARP1, CDC25B, and PTGS2, were taken as inputs. The ligands and the proteins were isolated, GAFF forcefield was applied to the former, and amber99sb-ildn forcefield was applied to the latter. After producing the protein and ligand topologies, the complex was remade and then solvated using spce as the water model. An octahedron 0.8 nm away from the system was used as the solvent box, with Na and Cl ions serving to neutralize any protein charges. Energy minimization was then conducted with 5000 steps. Afterward, NVT and NPT were conducted, each with 5000 steps over a time span of 10 ps.

#### 4.7.2. Production Molecular Dynamics

Official molecular dynamics was conducted using Gromacs 2024.4 [42] over a span of 100 ns corresponding to 50,000,000 steps (2 fs per step). Statistical analysis of the structural stability analysis involving the RMSD, radius of gyration, RMSF, and total number of hydrogen bonds was conducted using the Kolmogorov–Smirnov test [43] to determine the nature of the distributions. Cliff’s Delta [44] and Mann–Whitney U testing [45] were then used to evaluate the degree of difference and the shift in the median, respectively.

### 4.8. ADMET Analysis

SwissADME webserver (from http://www.swissadme.ch/) was used to evaluate the absorption, metabolism, and druglikeness (accessed on 9 March 2024) of the studied compounds [46]. SMILES were collected from PubChem entries, as shown in Table 6.

## 5. Conclusions

This study highlights the potential anti-H1N1 Influenza A activity of bioactive compounds extracted from *S. ardesiacus*, emphasizing their molecular interactions and inhibitory effects. Among the four major compounds isolated and characterized—1-acetyl-β-carboline, 1*H*-indole-3-carbaldehyde, Anthranilic acid, and Indole-3-carboxylic acid—1-acetyl-β-carboline demonstrated the strongest inhibitory activity, with an IC_50_ of 9.71 μg/mL, showcasing its potential as a lead compound for anti-influenza drug development. Network pharmacology analysis identified 186 genes associated with *S. ardesiacus* isolates and 194 genes related to H1N1 Influenza A, with a high confidence threshold of 0.9. Key proteins—CDC25B, PARP1, and PTGS2—were identified through topological analysis and linked to catalytic activity, inflammatory response, and cell cycle regulation, as highlighted by Gene Ontology enrichment. KEGG pathway analysis further revealed that serotonergic synapse, steroid hormone biosynthesis, and apoptosis are key pathways influenced by these compounds. Molecular docking demonstrated that 1-acetyl-β-carboline exhibited binding affinities comparable to Tamiflu, the positive control drug. LibDock scores for 1-acetyl-β-carboline with CDC25B, PARP1, and PTGS2 were 81.89, 77.49, and 89.21, respectively, compared to Tamiflu’s scores of 84.34, 86.13, and 91.29. These results suggest that *S. ardesiacus* isolates, particularly 1-acetyl-β-carboline, have competitive inhibitory potential against H1N1 target proteins, highlighting their promise as novel anti-influenza agents. By emphasizing the significance of 1-acetyl-β-carboline, this study sets the stage for further in vitro and in vivo investigations to validate its efficacy and safety. These findings underscore the potential of marine-derived microorganisms as a rich resource for innovative pharmaceutical development, addressing critical global health challenges like influenza.

## Figures and Tables

**Table 1:** Anti-influenza activity of isolates from *S. ardesiacus*.

Compound	IC_50_ (μg/mL)
1-acetyl-β-carboline (**1**)	9.71
1*H*-indole-3-carbaldehyde (**2**)	-
Anthranilic acid (**3**)	82.06
Indole-3-carboxylic acid (**4**)	81.49

**Table 2:** The top 3 Gene Ontology terms of MF, BP, CC, and KEGG pathways.

Source	NO.	Term Name	*P*_adj_ *
GO MF	1.	Catalytic activity	3.961 × 10^−51^
	2.	Oxidoreductase activity	1.328 × 10^−25^
	3.	Catalytic activity, acting on a protein	3.970 × 10^−23^
GO BP	1.	Response to chemical	3.154 × 10^−52^
	2.	Response to organic substance	2.615 × 10^−49^
	3.	Response to lipid	1.942 × 10^−32^
GO CC	1.	Cytoplasm	7.661 × 10^−21^
	2.	Cytosol	8.938 × 10^−14^
	3.	Somatodendritic compartment	2.459 × 10^−13^
KEGG	1.	Serotonergic synapse	9.785 × 10^−14^
	2.	Steroid hormone biosynthesis	1.024 × 10^−11^
	3.	Apoptosis	5.850 × 10^−11^

* The y-axis of enrichment *p*-values is in a negative log_10_ scale. Gene Ontology (GO); Molecular Function (MF); Biological Process (BP); Cellular Component (CC); Kyoto Encyclopedia of Genes and Genomes (KEGG).

**Table 3:** LibDock scores.

Compounds	CDC25B (4WH7)	PARP1 (4RV6)	PTGS2 (5KIR)
1-acetyl-β-carboline (**1**)	81.89	77.49	89.21
1*H*-indole-3-carbaldehyde (**2**)	67.34	59.19	73.36
Anthranilic acid (**3**)	60.44	55.44	70.56
Indole-3-carboxylic acid (**4**)	74.56	65.38	79.47
Tamiflu *	84.34	86.13	91.29

*, positive control.

**Table 4:** The binding interaction between key targets and 1.

	CDC25B (4WH7)	PARP1 (4RV6)	PTGS2 (5KIR)
Conventional Hydrogen Bond	TYR428, SER549	ASP770, ARG878	TYR385, SER530
van der Waals	PRO444, ARG447, ARG479, MET483, PHE543, ARG544, ARG548, TRP550	VAL773, GLN875, ILE879, ALA880, PRO881, TYR889	LEU352, TYR355, LEU359, LEU384, TRP387, SER353, LEU531, PHE518
Amide-Pi Stacking	N.A.	N.A.	GLY526
Pi-Alkyl	LEU445	ARG878	VAL349, VAL523, ALA527
Pi-anion	GLU446, ARG482	ASP766, ARG878	N.A.
Pi-Donor Hydrogen Bond	ARG548	N.A.	N.A.
Pi-Sigma	THR547	LEU769	N.A.
Pi-Sulfur	N.A.	N.A.	MET522
Pi-cation	GLU446, ARG482	ASP766, ARG878	N.A.

N.A.: Not relevant.

**Table 5:** The Binding interaction between key targets and Tamiflu.

	CDC25B (4WH7)	PARP1 (4RV6)	PTGS2 (5KIR)
Conventional Hydrogen Bond	LYS399, MET505	ILE879, ARG878	SER119
Carbon Hydrogen Bond	ASP397	ARG878	PRO86, ARG120
van der Waals	GLU377, LEU378, ILE379, GLY380, PHE386, CYS484, ARG492, PRO503, GLU504, TYR506, ILE507	LYS703, GLN707, ASP766, ASP770, GLN875, ALA880, GLU883	LYS83, PRO84, VAL89, LEU93, TYR115, VAL116, TYR355, PHE470, ARG513, GLU524
Alkyl	TYR382, LEU398, ARG485, ARG488	ILE706, TYR710, LEU769, VAL773, PRO881.	HIS90, LEU123, MET471, VAL523.
Pi-Alkyl	TYR382, LEU398, ARG485, ARG488	ILE706, TYR710, LEU769, VAL773, PRO881	HIS90, LEU123, MET471, VAL523

N.A., Not relevant.

**Table 6:** Ligands and positive control drugs for Anti-H1N1 Influenza A.

Compound	*CID	Structure
1-acetyl-beta-carboline	638667	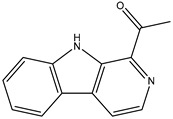
1*H*-indole-3-carbaldehyde	10256	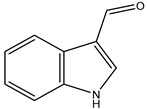
Anthranilic acid	227	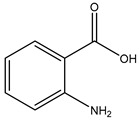
Indole-3-carboxylic acid	69867	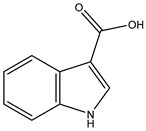
Tamiflu	78000	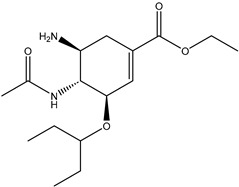

*CID: PubChem Compound Identifier.

**Table 7:** Coordinates and radii of each protein.

Name	Binding Spheres (X, Y, Z)			Radius
	X	Y	Z	
CDC25B (4WH7)	9.64	−8.13	−10.89	14
PARP1 (4RV6)	91.90	−16.98	134.36	14
PTGS2 (5KIR)	18.43	10.98	33.81	11

## Data Availability

The data that support the findings of this study are available from the corresponding author upon reasonable request.

## Supplementary Materials

The following supporting information can be downloaded at https://www.mdpi.com/article/10.3390/md23040149/s1, Figure S1. The ^1^H-NMR spectrum of Compound 1; Figure S2. The ^13^C-NMR spectrum of Compound 1; Figure S3. ESI-MS spectrum of Compound 1; Figure S4. The ^1^H-NMR spectrum of Compound 2; Figure S5. The ^13^C-NMR spectrum of Compound 2; Figure S6. ESI-MS spectrum of Compound 2; Figure S7. The ^1^H-NMR spectrum of Compound 3; Figure S8. The ^13^C-NMR spectrum of Compound 3; Figure S9. LCMS spectrum of Compound 3; Figure S10. The ^1^H-NMR spectrum of Compound 4; Figure S11. LCMS spectrum of Compound 4; Figure S12. RMSD values for PARP1 (A), CDC25B (B), and PTGS2 (C) complex with 1-acetyl-beta-carboline and Tamiflu as the test and reference ligands, respectively; Figure S13. Radius of gyration for PARP1 (A), CDC25B (B), and PTGS2 (C) complex with 1-acetyl-beta-carboline and Tamiflu as the test and reference ligands, respectively; Figure S14. Backbone RMSF for PARP1 (A), CDC25B (B), and PTGS2 (C) complex with 1-acetyl-beta-carboline and Tamiflu as the test and reference ligands, respectively; Figure S15. Total number of hydrogen bonds for PARP1 (A), CDC25B (B), and PTGS2 (C) complex with 1-acetyl-beta-carboline and Tamiflu as the test and reference ligands, respectively; Table S1. Summary statistics of the drug and reference molecular dynamics; Table S2. SwissADME results relevant to absorption; Table S3. Inhibitory potential to important metabolic enzymes; Table S4. Druglikeness evaluation of potential drug compounds and Tamiflu.

## Abbreviations

BCRC	Bioresource Collection and Research Center
BP	Biological Process
CC	Cellular Component
COX-1	Cyclooxygenase-1
COX-2	Cyclooxygenase-2
DMEM	Dulbecco’s Modified Eagle Medium
TLC	Thin-layer chromatography
FBS	Fetal bovine serum
FIRDI	Food Industry Research and Development Institute
GO	Gene Ontology
KEGG	Kyoto Encyclopedia of Genes and Genomes
MDCK	Madin-Darby Canine Kidney
MF	Molecular Function
PPI	Protein–Protein Interaction
TPCK	L-1-tosylamido-2-phenylethyl chloromethyl ketone
