# Supplementary material for: In Vitro and In Silico Studies on the Anti-H1N1 Activity of Bioactive Compounds from Marine-Derived Streptomyces ardesiacus
Source: Mar Drugs. 2025 Mar 29;23(4):149. doi: 10.3390/md23040149 (PMC12028705; doi:10.3390/md23040149)
Supplement: Supplementary file 1 [file marinedrugs-23-00149-s001.zip › marinedrugs-3510444-supplementary.pdf]

Supplementary Information

Contents

Figure S1. The ^{1}H-NMR spectrum of compound 1

Figure S2. The ^{13}C-NMR spectrum of compound 1

Figure S3. ESI-MS Spectrum of compound 1

Figure S4. The ^{1}H-NMR spectrum of compound 2

Figure S5. The ^{13}C-NMR spectrum of compound 2

Figure S6. ESI-MS Spectrum of compound 2

Figure S7. The ^{1}H-NMR spectrum of compound 3

Figure S8. The ^{13}C-NMR spectrum of compound 3

Figure S9. LCMS Spectrum of compound 3

Figure S10. The ^{1}H -NMR spectrum of compound 4

Figure S11. LCMS Spectrum of compound 4

Figure S12. RMSD values for PARP1 (A), CDC25B (B), and PTGS2 (C) complex with 1-acetyl-beta-carboline and Tamiflu as the test and reference ligands respectively

Figure S13. Radius of gyration for PARP1 (A), CDC25B (B), and PTGS2 (C) complex with 1-acetyl-beta-carboline and Tamiflu as the test and reference ligands respectively

Figure S14. Backbone RMSF for PARP1 (A), CDC25B (B), and PTGS2 (C) complex with 1-acetyl-beta-carboline and Tamiflu as the test and reference ligands respectively

Figure S15. Total number of hydrogen bonds for PARP1 (A), CDC25B (B), and PTGS2 (C) complex with 1-acetyl-beta-carboline and Tamiflu as the test and reference ligands respectively

Table S1. Summary statistics of the drug and reference molecular dynamics

Table S2. SwissADME results relevant to absorption

Table S3. Inhibitory potential to important metabolic enzymes

Table S4. Druglikeness evaluation of potential drug compounds and Tamiflu

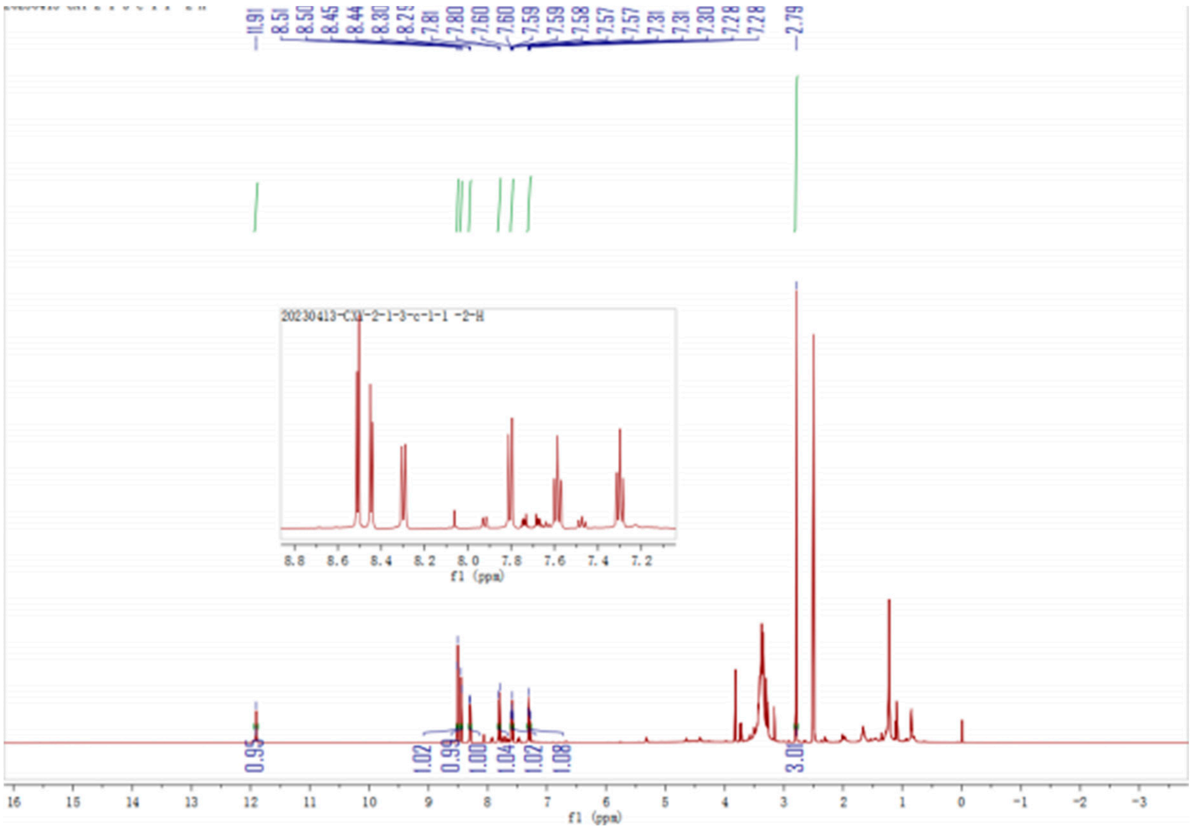

Figure S1. The ^{1}H-NMR spectrum of compound 1

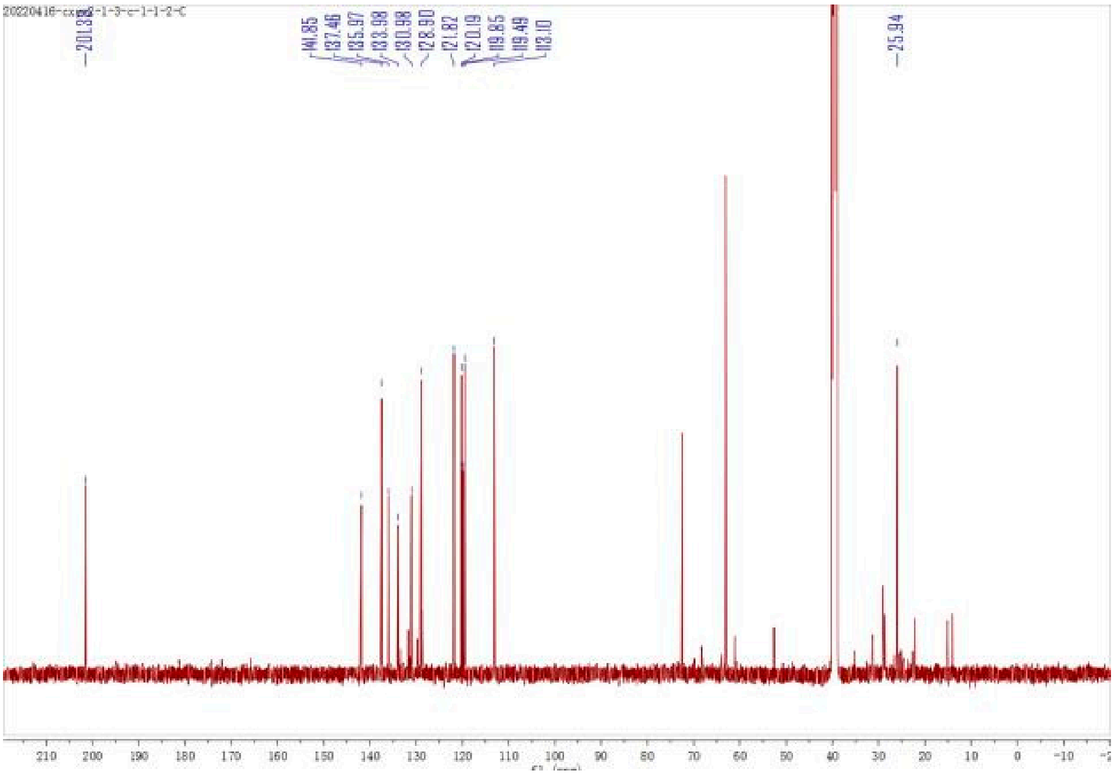

Figure S2. The ^{13}C-NMR spectrum of compound 1

ESI-MS Spectrum of compound **1**

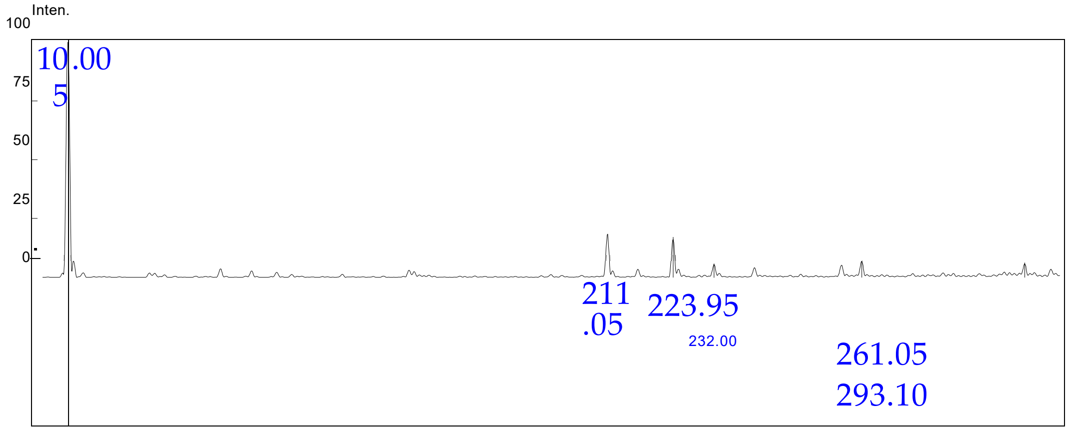

Figure S3. ESI-MS Spectrum of compound 1

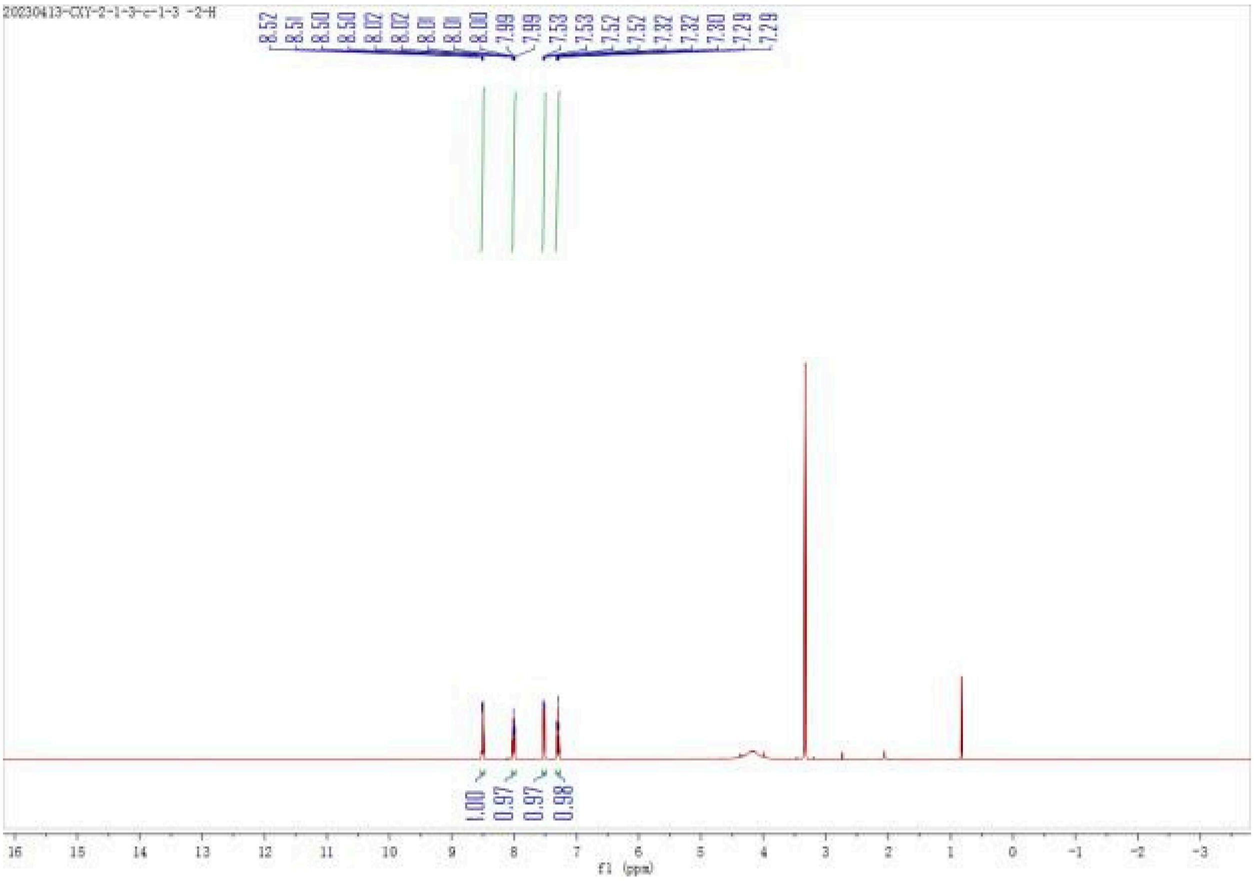

Figure S4. The ^{1}H-NMR spectrum of compound 2

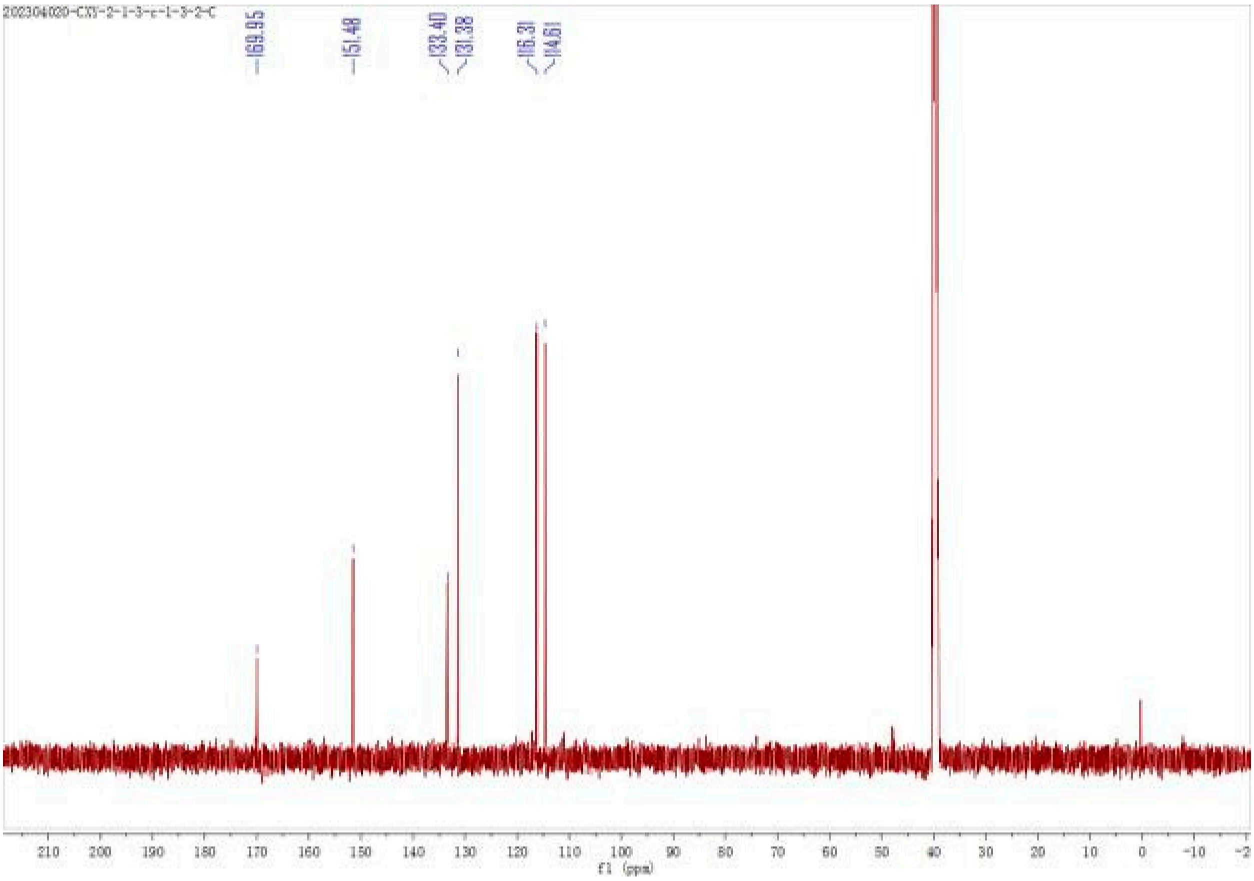

Figure S5. The ^{13}C-NMR spectrum of compound 2

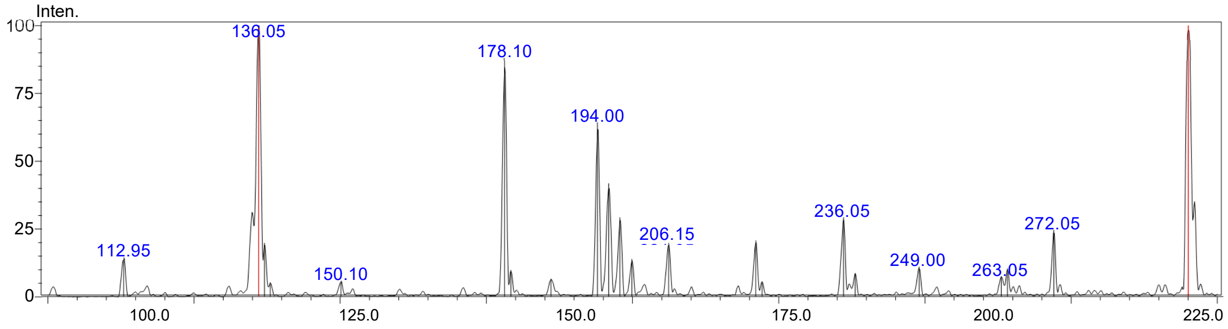

Figure S6. ESI-MS Spectrum of compound 2

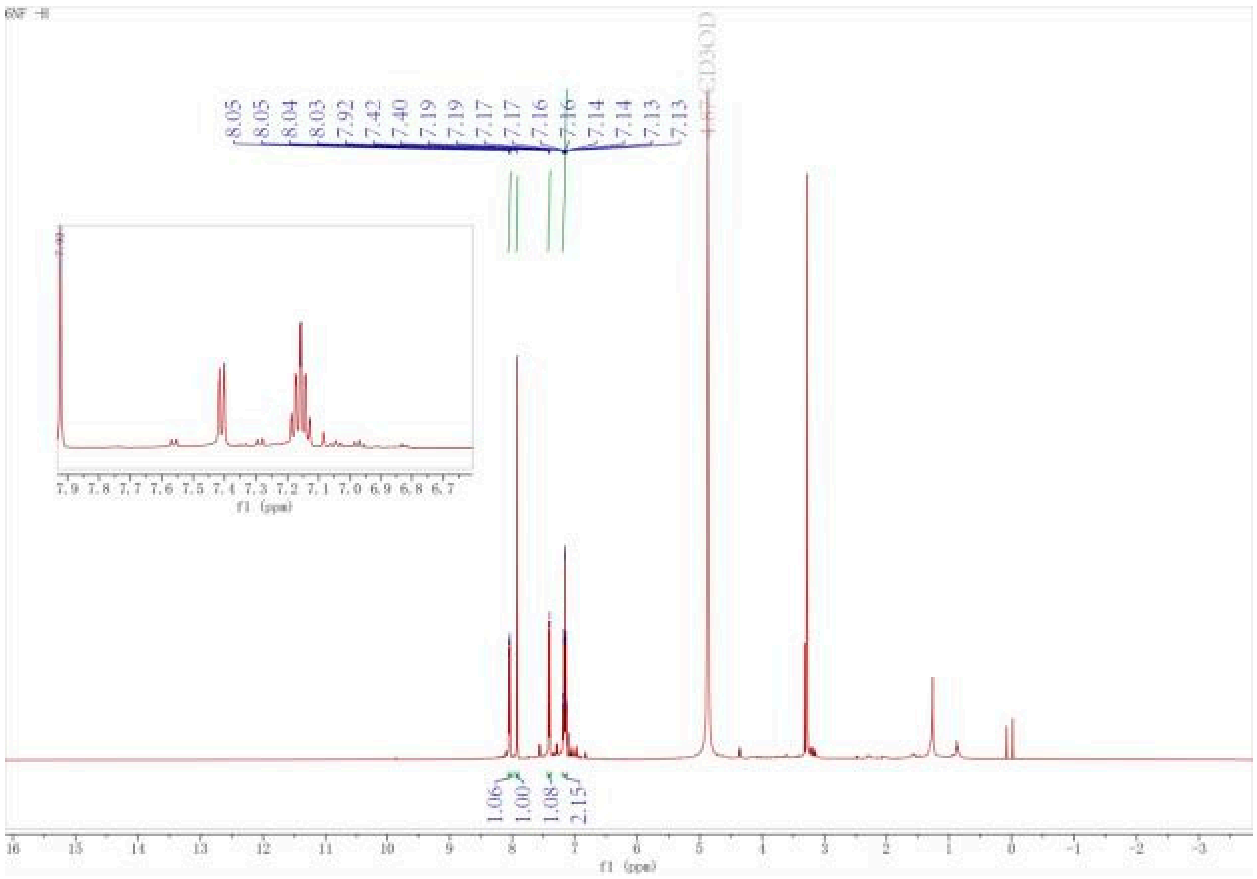

Figure S7. The ^{1}H-NMR spectrum of compound 3

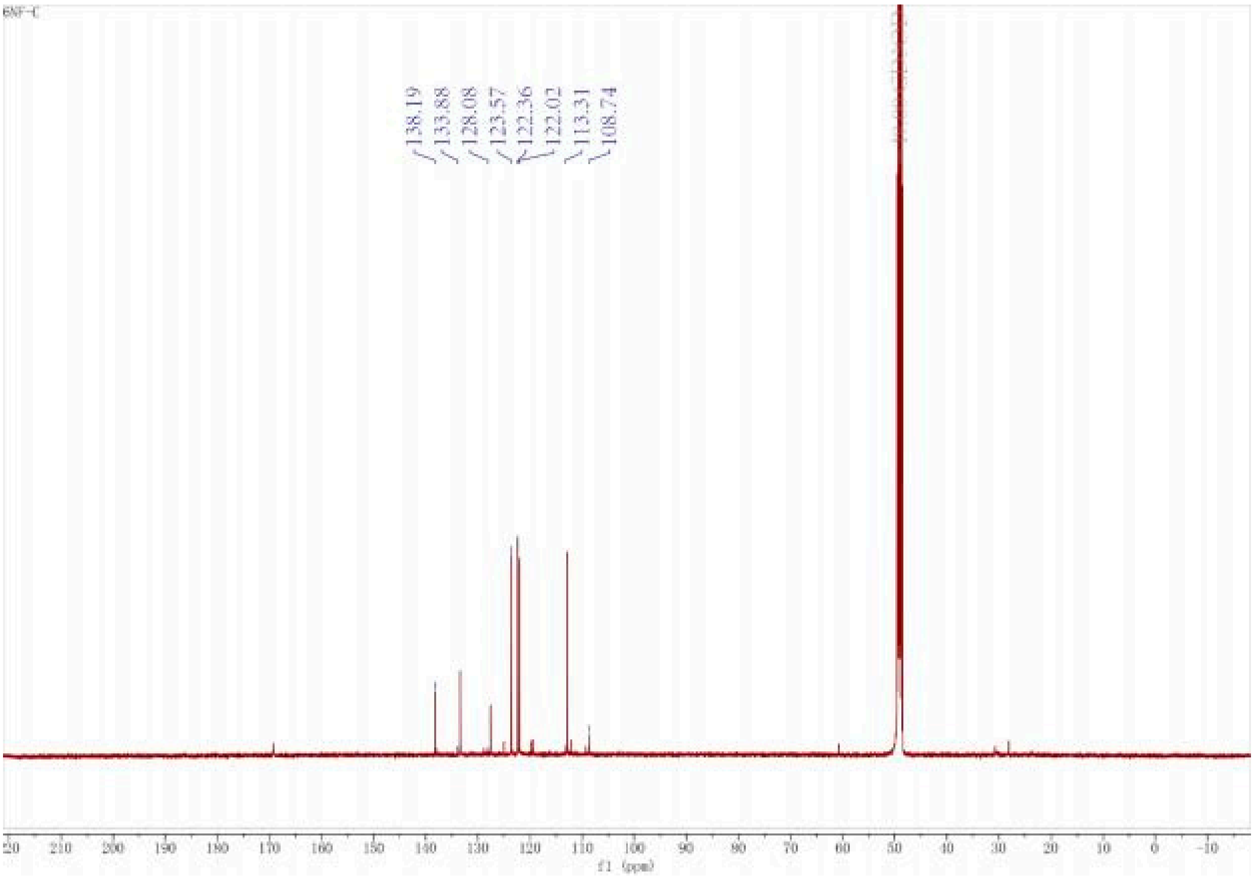

Figure S8. The ^{13}C-NMR spectrum of compound 3

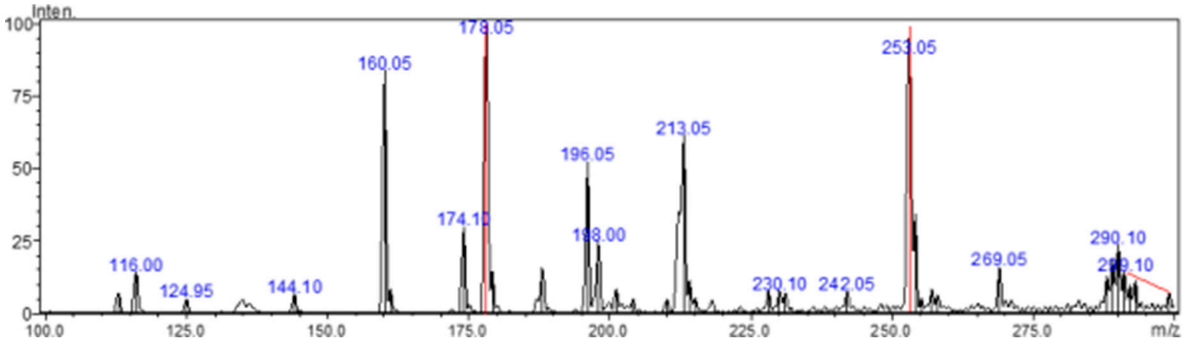

Figure S9. LCMS Spectrum of compound 3

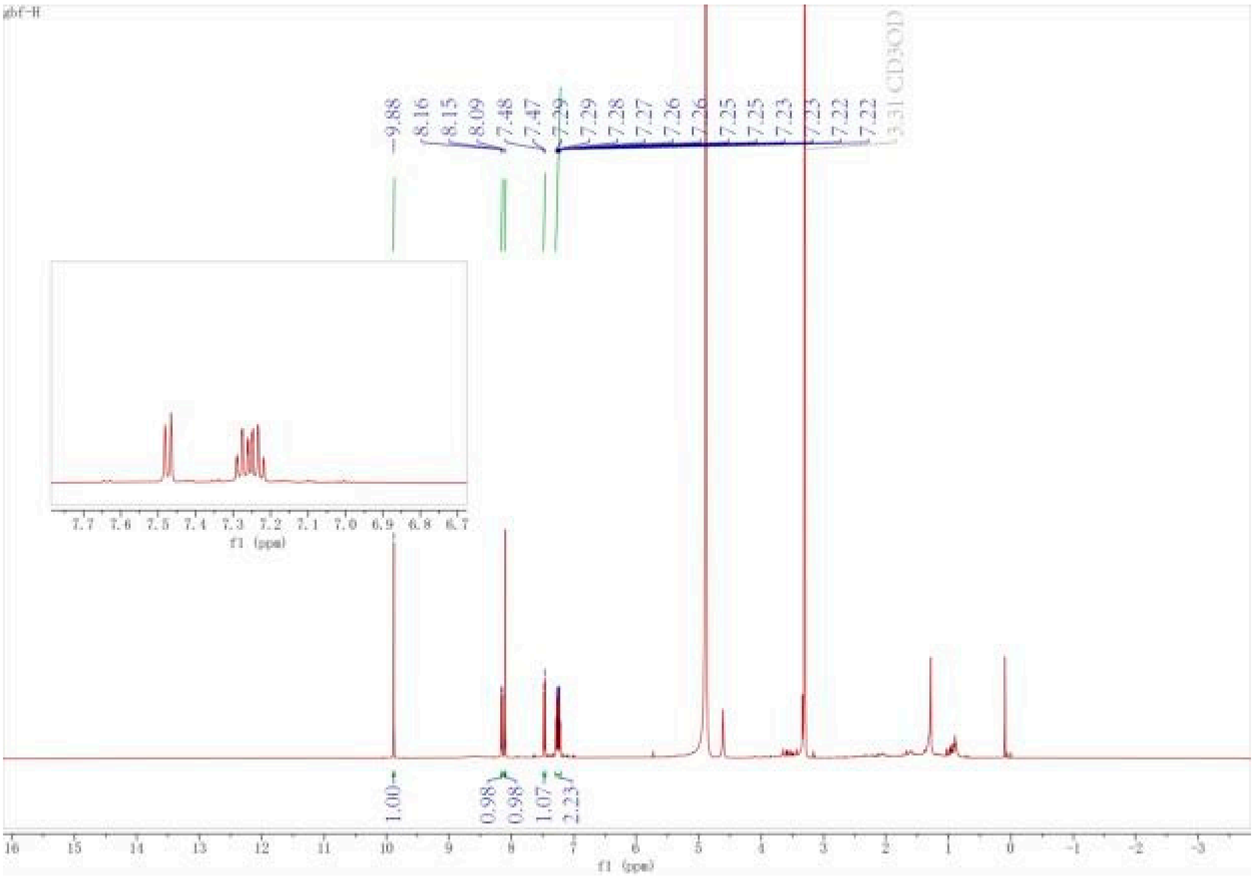

Figure S10. The ¹H -NMR spectrum of compound 4

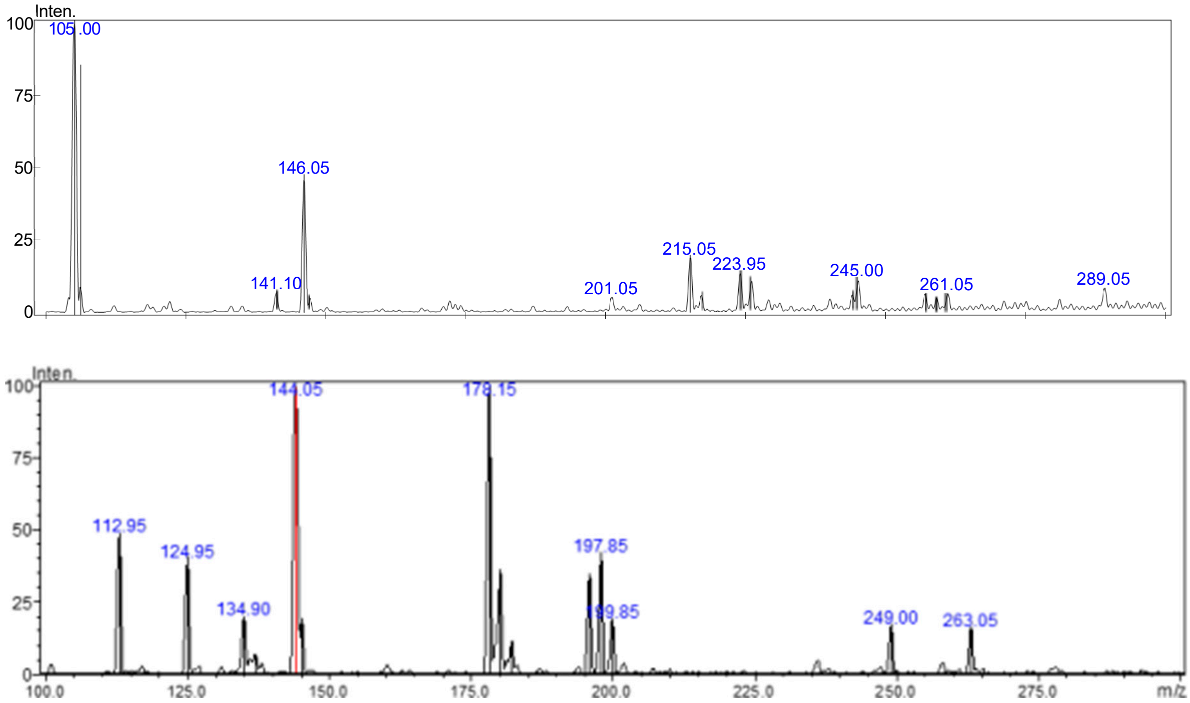

Figure S11. LCMS Spectrum of compound 4

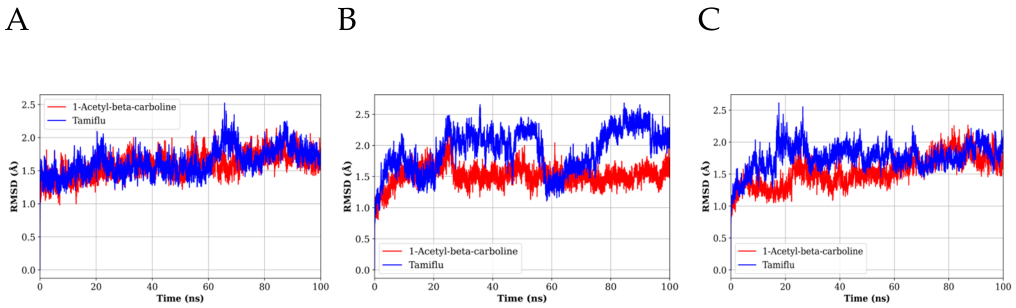

Figure S12. RMSD values for PARP1 (A), CDC25B (B), and PTGS2 (C) complex with 1-acetyl-beta-carboline and Tamiflu as the test and reference ligands respectively.

A B C

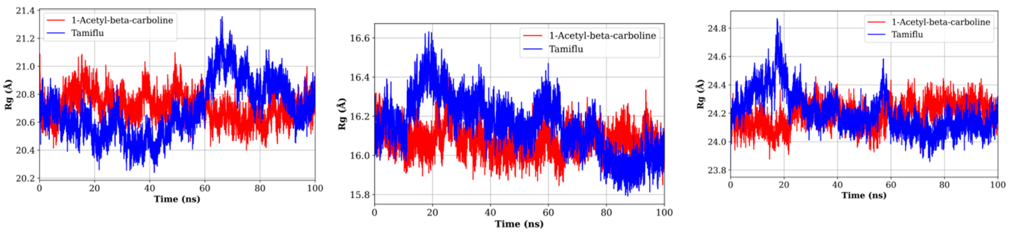

Figure S13. Radius of gyration for PARP1 (A), CDC25B (B), and PTGS2 (C) complex with 1-acetyl-beta-carboline and Tamiflu as the test and reference ligands respectively.

A B C

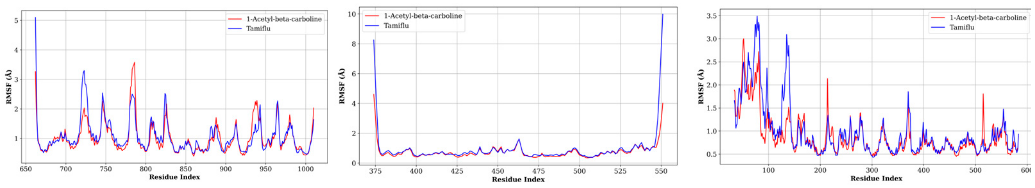

Figure S14. Backbone RMSF for PARP1 (A), CDC25B (B), and PTGS2 (C) complex with 1-acetyl-beta-carboline and Tamiflu as the test and reference ligands respectively.

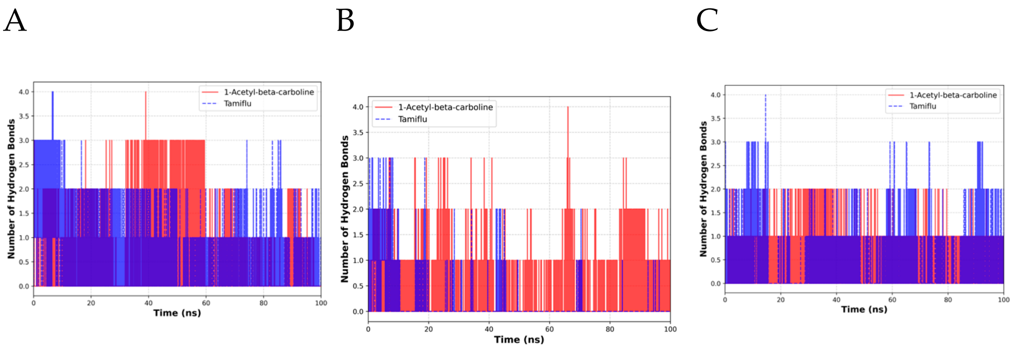

Figure S15. Total number of hydrogen bonds for PARP1 (A), CDC25B (B), and PTGS2 (C) complex with 1-acetyl-beta-carboline and Tamiflu as the test and reference ligands respectively.

Table S1. Summary statistics of the drug and reference molecular dynamics.

RMSD

					**Reference**		**Drug**		
**Cliff's Delta**[5]	**MW p-value**[4]	**MW Stat**[3]	**KS p-value**[2]	**KS Stat**[1]	**Median**	**Mean**	**Median**	**Mean**	**Receptor**
-0.09	0	45728096.5	0	0.09	1.61	1.63	1.59	1.59	PARP1
-0.68	0	15971845.5	0	0.62	1.98	1.92	1.52	1.52	CDC25B
-0.55	0	22622389	0	0.45	1.77	1.76	1.53	1.54	PTGS2

Radius of gyration

					**Reference**		**Drug**		
**Cliff's Delta**[5]	**MW p-value**[4]	**MW Stat**[3]	**KS p-value**[2]	**KS Stat**[1]	**Median**	**Mean**	**Median**	**Mean**	**Receptor**
0.14	0	57065497.5	0	0.23	20.66	20.69	20.71	20.71	PARP1
-0.36	0	31815291	0	0.36	16.16	16.15	16.07	16.08	CDC25B

0	0.6	50224971.5	0	0.14	24.18	24.21	24.2	24.19	PTGS2

RMSF

					Reference		Drug		
**Cliff's Delta[5]**	**MW p-value[4]**	**MW Stat[3]**	**KS p-value[2]**	**KS Stat[1]**	**Median**	**Mean**	**Median**	**Mean**	**Receptor**
-0.06	0.21	56874	0.24	0.08	0.9	1.05	0.87	1.01	PARP1
-0.14	0.02	13628	0.02	0.16	0.68	0.94	0.64	0.76	CDC25B
-0.09	0.01	138562.5	0.06	0.08	0.77	0.98	0.73	0.88	PTGS2

Total number of hydrogen bonds

					Reference		Drug		
**Cliff's Delta[5]**	**MW p-value[4]**	**MW Stat[3]**	**KS p-value[2]**	**KS Stat[1]**	**Median**	**Mean**	**Median**	**Mean**	**Receptor**
0	0.31	1245849588	0	0.03	0	0.63	0	0.6	PARP1
0.16	0	1455447500	0	0.18	0	0.2	0	0.34	CDC25B

0.14	0	1426471059	0	0.17	0	0.5	1	0.62	PTGS2

[1]Kolmogorov-Smirnov statistic, [2]Kolmogorov-Smirnov p-value, $p < 0.05$ indicates significant difference in the distributions, [3]Mann-Whitney U test statistic, [4]Mann-Whitney U test p-value, $p < 0.05$ indicates significant difference in the medians, [5]Cliff's delta, positive values indicate reference values are higher than the drug values.

Table S2. SwissADME results relevant to absorption.

**log Kp (cm/s)**	**Pgp substrate**	**BBB permeant**	**GI absorption**	**Molecule**
-5.93	No	Yes	High	1-acetyl-β-carboline (**1**)
-5.82	No	Yes	High	1*H*-indole-3-carbaldehyde (**2**)
-6.28	No	Yes	High	Anthranilic acid (**3**)
-5.87	No	Yes	High	Indole-3-carboxylic acid (**4**)
-11.13	Yes	No	Low	Tamiflu

Table S3. Inhibitory potential to important metabolic enzymes.

CYP3A4	CYP2D6	CYP2C9	CYP2C19	CYP1A2	Molecule
Yes	No	No	No	Yes	1-acetyl-β-carboline (**1**)
No	No	No	No	Yes	1*H*-indole-3-carbaldehyde (**2**)
No	No	No	No	No	Anthranilic acid (**3**)
No	No	No	No	Yes	Indole-3-carboxylic acid (**4**)
No	No	No	No	No	Tamiflu

Table S4. Druglikeness evaluation of potential drug compounds and Tamiflu.

Alerts				Violations					
**Leadlikeness**	**Brenk**	**PAINS**	**BAS[1]**	**Muegge**	**Egan**	**Veber**	**Ghose**	**Lipinski**	**Molecule**
1	0	0	0.55	0	0	0	0	0	1-acetyl-β-carboline (**1**)
1	1	0	0.55	1	0	0	2	0	1*H*-indole-3-carbaldehyde (**2**)
1	1	0	0.85	1	0	0	3	0	Anthranilic acid (**3**)
1	0	0	0.85	1	0	0	1	0	Indole-3-carboxylic acid (**4**)
2	1	0	0.55	2	1	1	0	0	Tamiflu

[1]Bioavailability score
